# Action research in radiography: What it is and how it can be conducted

**DOI:** 10.1002/jmrs.8

**Published:** 2013-04-02

**Authors:** Zachary Munn, Alan Pearson, Zoe Jordan, Frederick Murphy, Diana Pilkington

**Affiliations:** 1 The Joanna Briggs Institute, Faculty of Health Sciences, The University of AdelaideAdelaide, South Australia, Australia; 2 Directorate of Radiography, School of Health Sciences, University of SalfordSalford, Lancashire, United Kingdom; 3 Magnetic Resonance Imaging Department, Royal Adelaide HospitalAdelaide, South Australia, Australia

**Keywords:** Action research, qualitative research, quality improvement

## Abstract

Action research is a form of research that investigates and describes a social or work situation with the aim of achieving a change which results in improvement. This article emphasizes the potential for action research to be a useful research method in radiography. A search was conducted to determine the extent to which action research has been utilized in radiography. Although action research has been used in a number of health-care settings, there are no published examples of action research being utilized in a clinical medical imaging department. Action research is discussed in detail, along with an example guide for an action research study. Action research has been identified as a useful way to affect change, to involve radiographers in the research process, and to introduce evidence-based practice to radiography.

## Introduction

There is a significant amount of literature that discusses the unique nature of medical imaging in the health-care system and the brief patient encounter that it entails. This short time frame for interaction coupled with the operation of sophisticated technology can often lead to patient care being overlooked.^1^–^6^ One strategy suggested to preserve the “humanity” in our profession is to conduct qualitative research.^7^ Historically, there has been an emphasis on quantitative research designs in medical imaging. However, these methods are not necessarily suitable to answer all questions related to radiography practice, in particular the “human” side of the profession, including the patient encounter and staff working relationships.^4^ This focus on quantitative research may stem from the historical dominance of the medical profession in medical imaging and the aim of medical imaging itself to quantify the disease process.^8^,^9^

In recent times, there has been a significant uptake of qualitative methods by radiographers and researchers in diagnostic imaging.^4^,^10^ This increase may stem from a number of influential articles discussing the need for a focus on and an increase in qualitative research, including the work of Dowd^7^ and Hammick^1^ late last century, and Adams and Smith^6^ and Ng and White^11^ early this century. There now exist examples of qualitative literature across a number of modalities within diagnostic imaging, such as magnetic resonance imaging (MRI),^12^ computed tomography (CT),^13^ ultrasound,^14^ bone densitometry,^15^ general radiography,^16^ and interventional radiography.^17^ In a recent systematic review looking at the experiences of patients undergoing medical imaging with either CT or MRI, 13 of the 15 studies were published after 2000,^4^ which displays the growth and sudden expansion of qualitative research in diagnostic imaging. The qualitative studies in this review highlighted the unique and diverse ways in which people experience high-technology medical imaging, and these experiences could not have been captured in as rich detail if the authors used quantitative studies.^4^

We can now see that both quantitative and qualitative approaches to inquiry are appropriate in medical imaging research, and both are important and complementary to each other. When planning a research project, the question being asked should direct the choice of the research approach. There is also scope to perform multimethod studies, which incorporate both qualitative and quantitative approaches, and may be useful to inform medical imaging professionals.^6^ However, it is imperative that these studies are not “mix and match research”^18^ (p. 191), but that there is congruence with the methodological approach for each method and that the research strategies used supplement each other.^18^ One such research design that may incorporate both quantitative and qualitative methodologies is action research.

### Action research

Action research is a form of critical inquiry based on the works of Kurt Lewin, a social psychologist whose early work focused on community action programmes in the United States of America during the 1940s.^19^–^21^ The approach taken by Lewin “combined generation of theory with changing the social system through the researcher acting on or in the social system” (p. 586).^21^ Lewin stated that research that produced nothing but books was insufficient, and believed that the research needed for social systems required action as a central component, which would emerge through the process of research.^22^ Action research is unlike traditional qualitative (or interpretive) studies, which can be viewed as a passive approach to research; action research can be seen as taking an activist approach with the end goal being action taking resulting in change.^23^ From these beginnings, action research has evolved over the years into many different types of action research, each with its own unique perspective.^24^ However, there do exist some key principals of action research common across the different approaches.

A number of definitions for action research have been put forward by numerous authors, which reflects the variety seen in the approaches defined as action research.^25^ Waterman et al.^25^ carried out a systematic review with the aim of providing a definition for action research and introduced their comprehensive definition with:

*Action research is a period of inquiry that describes, interprets and explains social situations while executing a change intervention aimed at improvement and involvement. It is problem-focused, context specific and future-oriented*.^25^

Action research is an inclusive research methodology, where the traditional model of the investigator studying or observing subjects does not necessarily apply. The action research investigator/researcher accepts that there exists not only a need to be aware of how people understand their actions and practice (such as in interpretive studies), but also a need to engage with them by forming a partnership to enable active change.^23^,^25^ There is no clear delineation between those conducting the research and the subjects (those being researched) as in traditional positivist study designs.^20^ This is exemplified in the terminology of action research, where those being researched are not necessarily called subjects, but participants.^26^

Action research is a complex, reflexive, and cyclical research methodology which cannot be reduced to a single method of inquiry, such as qualitative or quantitative methods, and it is often the case that multiple approaches to collecting and analyzing data are taken.^27^–^29^ Multiple approaches lead to triangulation, which allow a deeper understanding and a fuller and rounded picture of the construct under investigation, as it is viewed through a number of lenses and different data sets.^29^ By utilizing a number of different data collection methods, the credibility (and therefore trustworthiness)^10^ of the research can be improved, by complementing the limitations of one stated method with the strengths of another.^30^

In their book, *Action Research For Health And Social Care*, Hart and Bond^30^ outline a typology for action research, and describe four distinct approaches: experimental, organizational, professionalizing, and empowerment. Experimental action research is linked most closely with the work of early action researchers, which included Lewin's work and the use of a scientific approach to social problems.^30^ Organizational action research is used to address organizational issues, for example, staff absenteeism, and create productive working environments that are not resistant to change.^30^ The empowering approach focuses on anti-oppressiveness and working with vulnerable groups.^30^ The professionalizing approach, which may be particularly useful in radiography, “is informed by an agenda grounded in practice which also reflects the establishment of the new professions … to enhance their status on a par with the established professions, such as law and medicine, and to develop research-based practice” (p. 45).^30^

**Figure 1:**
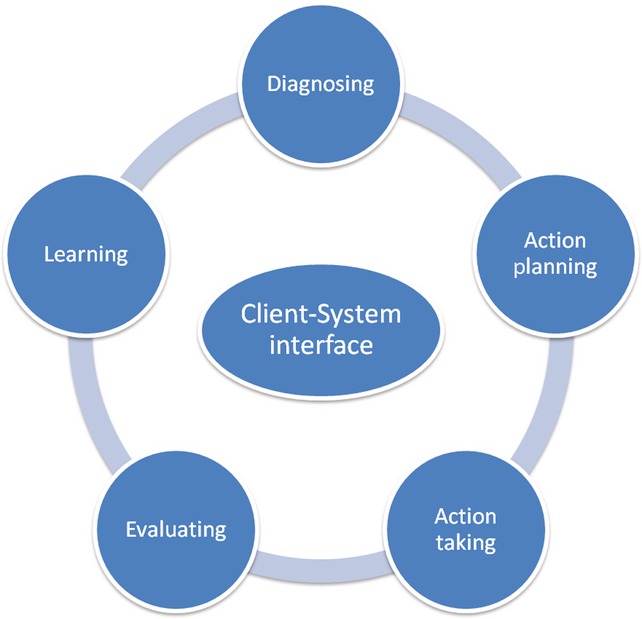
The cyclical process of action research as advocated by Susman and Evered.^21^

The conduct of action research should be guided by a methodological framework. The authors of this study plan to perform an action research study informed by the approach to action research advocated by Susman and Evered.^21^ Susman and Evered identified five phases, presented in a cyclical pattern, that are necessary in action research. These phases are diagnosing, action planning, action taking, evaluating, and specifying learning.^21^ At the centre of this cyclical process is the development of a client–system interface, which can inform all five phases (Fig. 1). This basic framework was chosen because it is conducive with the aims of the authors' project.

### Action research in radiography

In her polemic supporting of the importance of research in radiography, and specifically qualitative research, Hammick^1^ described the need for action research to be performed in radiography. Radiography is an emerging profession which faces low professional esteem and apathy due to low professional status, low public profile, and lack of professional recognition.^9^,^31^ The action research approach can make a positive contribution to the developing research base in radiography and contribute to the professionalization of radiography through the growth of professional knowledge.^31^ Action research in radiography can also encourage practitioners to be reflective in their practice and support the implementation of research into practice.^1^ Workplaces that promote critical thinking and reflection may then see enhanced clinical practice and improved health-care delivery as an outcome.^9^,^31^

This type of research, which involves researchers and practitioners, who can be one and the same, collaborating, may be used to investigate problems in need of solving.^1^ This is an essential feature of action research, although this should not be misconstrued as implying that there is something wrong in the department.^30^ Rather, this process will involve finding out what is currently happening in the department, “the real,” while comparing this to the “ideal,” which will emerge from discussions with those involved in the project. The gap between the real and ideal is where the problem or area for improvement will be identified.^30^

## Methods

To determine the uptake of action research in radiography, searches of MEDLINE (1996–2011) via Ovid were conducted using a number of key terms (Table 1). Only articles describing the use of action research in radiography were considered relevant.

**Table 1:** Search strategy

Search strategy	Results obtained	Relevant results
Search 1: (action research [keyword] or Health Services Research [MeSH]) and (Radiography [MeSH] or radiography [Keyword])	27	0
Search 2: (action research [keyword] or Health Services Research [MeSH]) and (Diagnostic Imaging [MeSH] or diagnostic imaging [Keyword])	46	0
Search 3: (action research [keyword] or Health Services Research [MeSH]) and (Diagnostic imaging [MeSH] or medical imaging [Keyword])	43	0

## Results

Despite Hammick's^1^ article positioning the need for action research to be performed in radiography, it has not been readily adopted (see Table 1).

Although there are numerous examples of action research being undertaken successfully in other areas of healthcare, no examples of its use in radiography were found via the MEDLINE search. However, a Google scholar search did present one example of action research being used in diagnostic imaging education by Palarm et al.,^32^ but not in radiography practice.

### Guide for radiography research

The authors plan to conduct an action research study in an MRI department to determine its feasibility in radiography and to assess whether it has the ability to improve practice in terms of patient care. Patient care is often overlooked in radiography,^1^–^6^ and despite the well-being of patients often being stated as the highest priority of radiographers, this is often truer in words than in practice.^33^ The proposal for this study is summarized in a guide form below, and may act as a resource for other radiographers who wish to conduct an action research study in their department, particularly if wishing to introduce a change in practice. Before conducting any research study, action researchers should be aware that the question is likely to change during the course of the project.^34^ This can occur as a result of the data collected, with this change becoming part of the outcome of the research and contributing to the discussion. Prior to conducting any research study, ethics advice and approval should be sought.

The first process prior to conducting action research is gaining access to the field or identifying a location where the research can be undertaken. This may be a challenging process in itself. Meetings will need to be held with the “professional gatekeepers” as described by Morton-Cooper,^34^ who are key people in the department and have the necessary influence to assist in bringing together and establishing the project. When negotiating access to the field, Morton-Cooper advises that the researcher should be modest and realistic in their requests, clear regarding resources, avoid stressful times, and offer something in return (i.e., a research bargain) for their efforts.^34^

### Establishing a client–system interface

Although different action research frameworks exist for the action researcher, the authors have found the Susman and Evered^21^ framework useful as it represents an action research process with a logical structure of five clear phases (see Fig. 1). However, prior to undertaking phase 1 (diagnosing), the client–system interface needs to be established by interaction with those who you will be working with during the action research project (i.e., staff in a radiology department). This approach, advocated by Pearson 1989 (cited in O'Brien)^35^ and followed by O'Brien, involves preliminary meetings and introductions with key personnel involved in the project. Discussions can focus on what the project hopes to achieve, timelines, and provide an opportunity to ask questions. The study design and approach should also be discussed and decided upon in partnership between the researcher and participants/co-researchers. For the author's project, this stage was particularly important as the lead researcher was coming in as an external agent to the workplace, and therefore time was required to establish themselves as a member of the team.^34^ As described by Wicks and Reason,^36^ “action research projects that are programmatic, designed and initiated from outside and imposed on participants … will result at best in … an intermediate group.”^36^ The success of action research projects can be determined by the initial discussions with co-researchers and staff, and the importance of opening communicative space (where participants can discuss issues or problems openly) has been stressed.^36^,^37^ Before the initial processes of action research (such as cycles of action and reflection) can occur, relationships with the appropriate people need to be established, the researcher is required to obtain legitimacy in the area, and an agreement “to engage in mutual inquiry” is required among all co-researchers/participants.^36^

#### Phase 1: Diagnosing

Following the development of a client–system interface, the first phase of data collection is conducted to identify or define a problem (the “gap” between the real and the ideal) currently existing in the area being investigated. Surveys/questionnaires, interviews, observation, and/or focus group interviews can be conducted at this stage, and the choice of method is dependent on access to the field and the feasibility of each method to address the problem. The aim of data collection at this stage is to elucidate the social norms and power structures within the action research setting and to identify any problems that currently exist in the department, known or unknown. An example problem that may be identified is that patients are receiving no information prior to their imaging or are being treated brusquely. Another may be that different professions are not functioning well together in an interdisciplinary team. A reflective journal with detailed notes can be kept throughout the process by the researchers to keep a record of their experiences throughout the project. All data analysis at this stage should be congruent with the collection methods used, such as thematic analysis for qualitative data, or statistical analysis for survey results.

#### Phase 2: Action planning

Once the data collected throughout phase 1 has been collated and analyzed, it should be presented back to all personnel working in the area. During this process, focus groups can be held to discuss the findings that emerged during phase 1, and actions can be planned collaboratively to be taken dependent on the results of these focus groups.

#### Phase 3: Action taking

The actions planned during phase 2 can then be implemented in the research setting.

#### Phase 4: Evaluating

After implementing the changes, a second period of data collection can be conducted using the same or similar methods as used previously, and analyzed accordingly.

#### Phase 5: Specifying learning

The results of the data should once again be fed back to all personnel, and the researchers can then determine the views of the participants regarding the change in their setting and the effect it has had on them and their work. General findings from the project can be identified and discussed. Following on from this, a second cyclical process may be undertaken if there is group consensus that adequate change has not been achieved.

## Conclusion

Although commonly performed in other health professions (such as nursing), a search has identified a lack of action research studies undertaken in the field of radiography. As radiographers may not be aware of benefits of this useful research method, this study provides a summary of the method and a guide for an action research study based on the author's experiences, which may act as a useful introduction to action research. In conclusion, this study describes that action research has been identified as a useful way to affect change in health-care settings, to involve radiographers in the research process, and to introduce evidence-based practice to radiography.
